# Characterization of the Expression Profile and Genetic Polymorphism of the Cellular Retinol-Binding Protein (CRBP IV) Gene in Erlang Mountainous Chickens

**DOI:** 10.3390/ijms14034432

**Published:** 2013-02-25

**Authors:** Hua-Dong Yin, Yan Wang, Zhi-Chao Zhang, Yi-Ping Liu, Shi-Yi Chen, Qing Zhu

**Affiliations:** Laboratory of Animal Genetic and Breeding, College of Animal Science and Technology, Sichuan Agricultural University, Ya’an, Sichuan 625014, China; E-Mails: yin881986@yahoo.com.cn (H.-D.Y.); wangyan519723614@yahoo.com.cn (Y.W.); zzchao5515@163.com (Z.-C.Z.); liuyp578@yahoo.com (Y.-P.L.); chensysau@163.com (S.-Y.C.)

**Keywords:** *CRBP IV* gene, mRNA expression, SNP, egg production, chicken

## Abstract

In this study, we cloned the coding sequence of chicken CRBP IV, quantified the mRNA expression in Erlang Mountainous Chickens, and investigated a polymorphism in this gene and its association with egg production traits among 349 individuals. The cloned fragment contained a 384 bp open reading frame, which encoded a predicted protein of 127 amino acids and was highly conserved among species. Expression of CRBP IV mRNA was detected in all eight tissues (small intestine, heart, liver, kidney, oviduct, ovary, pituitary, and hypothalamus) at different ages (12, 24, 32 and 45 w). High expression was found in small intestine, pituitary, kidney and liver, whereas it was low in the heart (*p* < 0.05). The CRBP IV mRNA levels changed with age in the various tissues, and were highly expressed in all tissues at 32 w, except for the heart. We identified one nucleotide substitution (c. 826T>C) in the second exon, which caused an amino acid change (p. S49L). Genotypes (TT, TC and CC) had significant effects on the age at first egg (AFE), total eggs for 300 days (TE300) and highest continuous laying days (HCLD). The CC genotype would be genetically advantageous to improve egg production traits due to earlier AFE, more TE300, and longer HCLD.

## 1. Introduction

Vitamin A (retinol) and its active derivative (retinoic acid) are vital for proper embryonic development in vertebrates [1]. Retinoic acid (RA) is the ligand that binds to heterodimers of the retinoic acid receptor (RAR) and the retinoid X receptor (RXR). The RA-receptor complex then binds to retinoic acid response elements (RAREs) in regulatory regions and activates gene transcription. The activated genes are involved in regulating important molecular signals for various pathways that play essential roles in vision, growth, reproduction, cellular differentiation and proliferation in the developing embryo [2–4]. In the mammalian embryo, retinoids are derived from the maternal circulation through the placenta. In contrast, oviparous vertebrate embryos obtain retinoids and carotenoids from the egg yolk and oil globuli, which are accumulated during vitellogenesis [5]. Because retinoids and carotenoids can not be synthesized by vertebrates, they must be fully provided from the diet in the form of vitamin A (primarily as retinol and retinyl-esters) or as provitamin A carotenoid [6]. Dietary retinol is absorbed in the small intestine, and up to 90% of whole body retinol is stored in the liver in the form of retinyl esters. Retinol is distributed between hepatocytes and hepatic stellate cells which are specific for retinyl ester storage [7].

Because of its chemical instability and low solubility in aqueous medium, retinol and its derivatives must be bound to specific proteins called retinoid-binding proteins (RBPs) [8]. Serum retinol-binding protein (RBP4) is the extracellular counterpart for retinol, and cellular retinol-binding proteins (CRBPs) are the intracellular counterparts for both retinal and retinol during the process of absorption, transport, and excretion [9]. Currently, there are four CRBPs that have been found, CRBP I, CRBP II, CRBP III and CRBP IV. These CRBPs not only antagonize retinoid hydrophobicity, but also affect the activity of enzymes involved in retinoid metabolism [10]. Conforti *et al.* identified a new member of the retinol-binding protein, Rbp7 (CRBP IV), in the slow Wallerian degeneration (*wld*^s^) mouse. Rbp7 is homologous to other members of the cellular retinoid-binding protein family, and to retinol-binding protein in particular, suggesting that *Rbp7* is likely to function in retinol sequestration and/or metabolism [11].

Native chickens are well known for their delicious and nutritious meat in China. However, lower reproduction and production performances are constraining the development of the indigenous chicken industry [12]. Reproduction is a complex process that is influenced by genetics and nutrition [13]. It was widely reported that a deficiency in vitamin A can lead to irregular ovulation, a decrease in egg production, and poor egg quality in the laying hen [14,15]. As a CRBP member, CRBP IV may play a role in absorption, transport, metabolism, and homeostasis of retinol and its derivatives. This makes CRBP IV a good candidate gene for enhancing reproductive traits in chickens. To test this hypothesis, we cloned and analyzed the entire coding region of the chicken CRBP IV gene, investigated the expression pattern of CRBP IV mRNA in various chicken tissues, and identified a genetic polymorphism and analyzed its association with chicken egg production traits.

## 2. Results

### 2.1. Molecular Cloning and Sequence of the Chicken CRBP IV Gene

We sequenced a PCR product encoding the Erlang mountainous chicken CRBP IV cDNA. The chicken CRBP IV cDNA contains an open reading frame (ORF) of 384 bp, which encodes a predicted protein of 127 amino acids (AA). Comparison of the obtained chicken CRBP IV AA sequence with those of *Gallus gallus* and six other species from GenBank (*Gallus gallus*, *Sus scofa*, *Mus musculus*, *Rattus norvegicus*, *Homo sapiens*, *Pan troglodytes*, *Bos taurus*, and *Canis lupus familiaris*) was made. We showed that the Erlang mountainous chicken AA sequence showed 98.4% identity to *Gallus gallus* and 57.5%–74.8% identity to mammals (Table 1).

**Figure 1:**
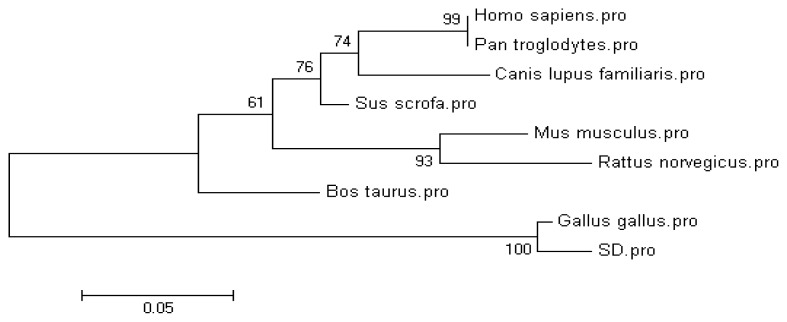
Phylogenetic tree constructed using Neighbor-joining method based on CRBP IV amino acid sequence from different species. Bootstrap support values based on 1000 replicates. SD = Erlang mountainous chicken.

A phylogenetic tree based on the amino acid sequence of chicken and other species was constructed as shown in Figure 1. The AA phylogenetic tree showed that the CRBP IV protein of vertebrates is divided into two main branches: mammalian and non-mammalian. The tree shows chicken CRBPIV to have a distant relationship with other mammals.

### 2.2. Ontogenic Expression of CRBP IV Gene in Chickens

**Figure 2:**
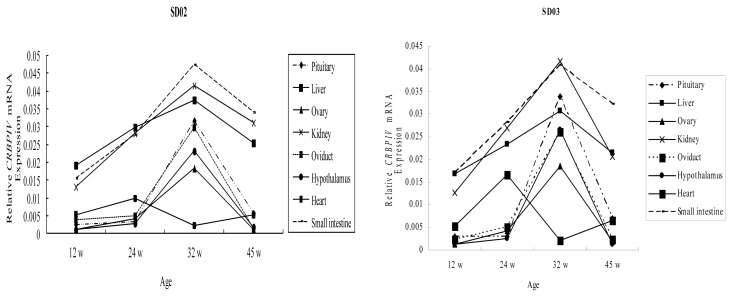
Ontogenic expression of the CRBP IV mRNA in different tissues collected from line SD02 and SD03 at different ages. W = weeks.

To determine if the CRBP IV mRNA expression shows tissue- and development-specific expression, we investigated its expression in eight tissues from two different chicken lines at four ages (12 w, 24 w, 32 w and 45 w). As shown in Figure 2, the ontogenic expression of CRBP IV mRNA showed similar profiles in all tissues of line SD02 and line SD03. Specifically, CRBP IV gradually increased in the pituitary, oviduct, hypothalamus and ovary tissue from 12 w to 24 w and then rapidly rose to its peak expression at 32 w. Gene expression then declined from 32 w to 45 w. In the kidney, liver, and small intestine of both lines, the expression of CRBP IV mRNA rapidly increased from 12 w to 32 w and subsequently declined to 45 w. In the heart, highest CRBP IV mRNA expression was found at 24 w and lowest expressions at 32 w in both lines.

### 2.3. Comparison of CRBP IV mRNA Expression among Different Tissues in Chickens

**Figure 3:**
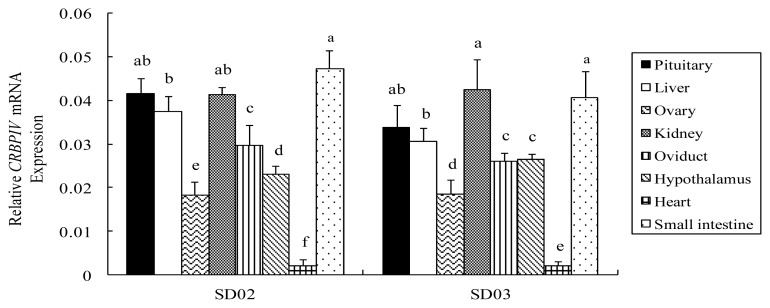
The relative mRNA of the *CRBP IV* gene in tissues collected from lines SD02 and SD03 at 32 weeks Different lowercase letters above the bars indicate significant differences (*p* < 0.05) within a line.

A comparison of CRBP IV mRNA expression in tissues at 32 w in line SD02 and SD03 is shown in Figure 3. In line SD02, CRBP IV mRNA level was greatest in the small intestine, pituitary, kidney, and liver, intermediate in the oviduct, hypothalamus, and ovary, and lowest in the heart (*p* < 0.05). A similar pattern of CRBP IV mRNA expression was also found in line SD03 (*p* < 0.05).

### 2.4. Identification of Genetic Variants in Chicken CRBP IV Gene

**Figure 4:**
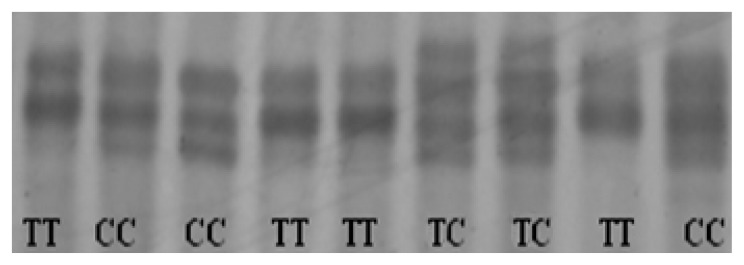
Electrophoregrams of the polymorphic patterns for c. 826T>C. The three genotypes of CC, TC, and TT were marked in lanes, respectively.

A PCR-based SSCP method was developed for screening for nucleotide substitutions in the three exons of the chicken CRBP IV gene. No polymorphism was shown based on SSCP banding pattern in the first and third exons. One SNP (c. 826T>C) in the second exon was verified by direct sequencing the polymorphic fragment based on the SSCP banding pattern (Figure 4). This polymorphism caused an amino acid change (p. S49L).

### 2.5. Genotype Frequencies and Association Analysis with Egg Production Traits

The genotypes and alleles of the identified SNP in the CRBP IV gene were analyzed, and results are shown in Table 2. For SNP of c. 826T>C, the allele T (average allele frequency = 63.57%) was predominant due to its high frequency compared to allele C (36.4%) in all populations. Three genotypes were determined with the average frequencies of TT (38.6%), TC (50.0%) and CC (11.4%) in 349 samples. Both line SD02 and line SD03 did not deviate from Hardy-Weinberg equilibrium according to the Chi-square test. The average polymorphism information content (PIC) of c. 826T>C was 0.356. The PIC for SD02 was 0.354 and the PIC for SD03 was 0.357.

The results about the GLM analysis of the association between the CRBP IV gene polymorphism and lines for egg production are summarized in Table 3. The least square mean analysis revealed that there was positive association of genotypes on c. 826T>C with AFE, TE300, and HCLD (*p* < 0.05). The CC genotype had the earliest AFE (169.3 ± 10.0 d) and the longest HCLD (9.7 ± 3.8 d). There was no significant difference for these two traits between TT and TC genotypes. For TE300, the CC genotype (85.6 ± 20.4 eggs) was greater than the TT genotype (77.9 ± 18.2 eggs) (*p* < 0.01) and the TC genotype (81.1 ± 16.9 eggs) was greater than the TT genotype (77.9 ± 18.2 eggs) (*p* < 0.05). The other five egg production traits were similar between lines SD02 and SD03, with the exception of AFE where SD02 was lower (169.8 ± 10.2) than SD03 (176.8 ± 10.9) (*p* < 0.05).

## 3. Discussion

Numerous studies have established vitamin A to be essential for reproduction, growth, and embryonic development [16]. Furthermore, CRBPs are necessary intracellular factors in the absorption, transport, and metabolism of vitamin A [17]. CRBP I, CRBP II, CRBP III and CRBP IV have been widely researched in human, mice, and other animals [18–20], but there is little knowledge about their functions in poultry, this is especially true for the CRBP IV gene. Understanding the tissue distribution and genetic variation of CRBP IV may contribute to identifying the effect it has on reproduction in chickens. Therefore, we cloned the cDNA for CRBP IV, characterized the mRNA expression profile and investigated the genetic association of a polymorphism with reproduction traits in Erlang Mountainous Chicken, a local breed, for the dual purpose of egg and meat in Sichuan.

In this study, we determined the Erlang mountainous cDNA sequence of chicken CRBP IV gene. The high similarity of CRBP IV AA among different species and the conserved domain of chicken CRBP IV strongly suggest functional conservation of this gene in vertebrates.

For the Erlang mountainous chickens used in this study, market date is 12 weeks, the average age at first egg is 24 w, peak egg production is around 32 w, and start of the end of egg production is at 45 w. The expression of CRBP IV mRNA was determined at four time points. At 32 w, all tissues except heart had the highest CRBP IV mRNA. Low expression was found at 12 w and 45 w. This may be due to the necessity of vitamin A at these time points. Vitamin A is highly required during peak lay (32 w) and not as important at other times [21]. This can also be the reason why the expression of CRBP IV rose from 24 w to 32 w and dropped thereafter.

CRBP IV mRNA was expressed in all of the examined tissues, but the expression pattern was not similar to other species. Human CRBP IV mRNA was expressed widely in many tissues with the greatest abundance in the adult kidney, and also highly expressed in adult heart, adult transverse colon, fetal heart, and fetal spleen [22]. The mRNA expression level of porcine CRBP IV was highest in the liver, followed by spleen, large intestine, and lymph node. Expression was weak in the heart, lung, intestine, kidney, and fat [23]. The house mouse CRBP IV mRNA was highly expressed in white adipose tissue and mammary gland, but low in heart, kidney, and brain [11]. In our study, CRBP IV was highly expressed in the small intestine and liver, which is consistent with the report that retinol is absorbed in the small intestine and stored in liver [24]. Why CRBP IV mRNA was abundant in kidney may also be due to vitamin A metabolism being regulated by kidneys [25]. Because vitamin A plays a very important role in the hypothalamus-pituitary-ovary axis [26,27], high CRBP IV expression in pituitary, hypothalamus, ovary, and oviduct was expected.

Among the three exons of chicken CRBP IV gene, only one non-synonymous nucleotide substitution (c. 826T>C) was found in the second exon. In contrast to the low genetic diversity throughout the coding sequence, the identified SNP had high polymorphism in the tested population with the average polymorphism information content of 0.356. The PIC is a significant parameter in evaluating the genetic variation and has three gradations: highly informative (PIC > 0.5), medium polymorphic (0.5 > PIC > 0.25), and slight variation (PIC < 0.25). In our study, the SNP of c. 826T>C was a medium polymorphic locus, which suggested that these two lines have better selective space for breeding.

Because the nucleotide substitution of c. 826T>C caused an AA change (p. S49L), to investigate the possible functional change from this mutation, we analyzed the correlation of CRBP IV genotypes with egg production traits. The least square mean analysis revealed that there was a positive association of genotypes at this SNP with AFE, TE300, and HCLD. Among them, the CC genotype had better egg production performances with earlier AFE, more TE300 and longer HCLD, and would be genetically advantageous in improving egg production traits. This result was in accordance with the function of CRBP IV protein on reproduction and supported our hypothesis that CRBP IV can be a candidate gene in selecting quality egg production performances. Many function genes, such as prolactin (*PRL*) [28], prolactin receptor (*PRLR*) [29], gonadotrophin releasing hormone receptor (*GnRHR*) [30], and growth hormone (*GH*) [31], have been positively associated with laying production parameters in chicken. However, to our knowledge, this is the first report to study the SNP distribution of the CRBP IV gene and its association of genotypes with egg production traits in chickens. Additionally, the main limitation of our study was the small number of samples, especially chickens, with the CC genotype. Further large-scale research should be conducted to investigate the effects of this SNP and verify its function on egg production traits in a larger chicken population.

It has been reported that laying production traits are affected by genetic, nutritional, and environmental factors [32–34]. Because all of the experimental chickens were fed under the same feeding conditions, we only analyzed the effect of lines on egg production traits. The result showed that AFE was affected not only by genotype, but also by lines. The effect of genotypes on traits may be limited by genetic background [35]. This illustrates that it is important to understand the genetic relationships among individuals, such as using neutral markers like microsatellite markers [36], before future application of marker-assisted selection plans.

## 4. Experimental Section

### 4.1. Chicken Populations and Trait Measurement

Erlang mountainous chickens are selecting by Sichuan Agricultural University and Ya’an Longsheng Animal Husbandry Co., Ltd., and have originated from a local chicken breed in Ya’an, Sichuan province. This includes two lines SD02 and SD03, which are widely adaptable, and have delicious and nutritious meat but show lower growth rate and productivity. The lines SD02 and SD03 were raised on an experimental farm at Sichuan Agricultural University (Ya’an, China) under the same conditions. For the mRNA expression studies, the eight tissues include small intestine, heart, liver, kidney, oviduct, ovary, pituitary, and hypothalamus, from 10 hens were collected at 12 w, 24 w, 32 w, and 45 w of age from each line. All fresh tissues were identified, excised, and rinsed in PBS, then frozen within cryogenic tubes in liquid nitrogen, and stored at −80 °C for mRNA analysis.

The total number of 349 hens was selected for association analysis between polymorphisms and egg production traits from two lines: SD02 (*N* = 188) and SD03 (*N* = 161). The wing vein blood samples were collected according to requirements of animal welfare of Sichuan Agricultural University, and store at −20 °C for DNA analysis.

Production traits were individually recorded from the age at first egg to egg production of 300 days, including age at first egg (AFE), body weight at first egg (BWFE), first egg weight (FEW), highest continuous laying days (HCLD), total eggs for 300 days (TE300), and average laying interval (ALI).

### 4.2. Total DNA Extraction, RNA Isolation and Reverse Transcription

The genomic DNA was extracted by a standard phenol/chloroform method. Total RNA was isolated from each tissue sample (approximately 50 mg fine powder) using TRIzol reagent (Invitrogen, Carlsbad, CA, USA) according to the manufacturer’s instructions. The RNA quality was determined by both native gel electrophoresis on 1.0% agarose gel and UV absorbance ratio at 260 and 280 nm, and quality was assessed with a Bio analyzer 2100 (Agilent Technologies, Santa Clara, CA, USA). All extracted RNA samples were stored at −80 °C. The complementary DNA was synthesized from 2 μg of total RNA by PrimeScript^®^ RT Master Mix Perfect Real Time (Takara, Dalian, China) following the manufacturer’s protocol.

### 4.3. Cloning and Sequencing of PCR Product

The primer pair (Table 1) was designed for amplifying the chicken CRBP IV gene according to the mRNA sequence of *Gallus gallus* (GenBank accession number XM_417606.2). 2 μL of the cDNA mixture was used as template in a 50 μL PCR reaction. After five minutes of denaturation at 94 °C, amplification was performed at 94 °C for 30 s, 55 °C for 40 s, 72 °C for two minutes for 32 cycles, with a final extension step of 10 min at 72 °C. PCR products were separated by electrophoresis on a 1.0% agarose gel and purified with a gel extraction kit (Watson Biomedical Inc., Shanghai, China). The purified PCR product was ligated into pMD-T vector (Invitrogen, Grand Island, NY, USA). Positive clones were selected for double-directional sequencing on an Applied Biosystem ABI 3100 Sequencer. The cDNA segments obtained from sequencing were edited and assembled with Editseq and Seqman programs in DNAStar software (version 9.0; DNASTAR, Inc., Madison, WI, USA).

### 4.4. Real-Time PCR, SNPs Identification and Association Analysis

The abundance of mRNA was determined on the real-time PCR thermal cycle instrument (Bio-Rad IQ-5, Hercules, CA, USA). Gene-specific primers (Table 4) for CRBP IV gene and endogenous reference gene (β-actin) were designed according to the published mRNA sequence of CRBP IV (GenBank accession number XM_417606.2) and β-actin (GenBank accession number NM_205518). PCR reactions performed in triplicate in a volume of 15 μL containing 1 μL of cDNA, 0.6 μL reverse and forward primers for each gene, 5.3 μL ddH_2_O and 7.5 μL SYBR®Prime Ex Taq™ II (Takara, Dalian, China). The PCR program was initial denaturation cycle for five minutes at 95 °C, followed by 35 cycles of 10 s at 95 °C, 30 s at 58 °C and 45 s at 72 °C, ending with 10 min at 72 °C. The standard curve was determined using pooled sample, and the specificity of PCR products was further confirmed by a melting curve analysis.

The PCR primer pairs for amplification of DNA sequence fragments were designed according to CRBP IV gene sequence of *Gallus gallus* (GenBank accession number NC_006108.2) and the cloned sequence of Erlang mountainous chicken. Three primer pairs were employed to amplify the three exons containing the coding sequence and investigated the genetic variation using the PCR-single stand conformation polymorphism (PCR-SSCP) method (Table 4).

The PCR amplification was conducted under the procedure: 95 °C for five minutes, 35 cycles at 94 °C for 45 s, (55~58) °C for 45 s, 72 °C for one minute and an extension at 72 °C for 10 min. The 10 μL reaction volume included 0.8 M (50 ng/μL) of template, 5 μL of 2×Taq PCR MasterMix (Bei jing TIAN WEI Biology Technique Corporation, Beijing, China), 3.4 μL of ddH_2_O and 0.4 μL of each primer (10 pmol/μL). To perform the SSCP analysis, the PCR products were denatured at 98 °C for 10 min and then placed on ice for five minutes. Electrophoresis was run at 10 V/cm on a 12% polyacrylamide gel for 9–10 h. Gels were stained using silver staining. Individual PCR-SSCP banding patterns were determined under visible light. Three DNA samples showing different patterns on SSCP gel were further amplified and purified, and then sequenced by Shanghai Yingjun Biology Technique Corporation (Shanghai, China).

### 4.5. Data Analysis

BLASTn program on NCBI was used to identify the cloned gene. Phylogenetic trees were constructed based on Kimura2–parameter (K2P) model by Mega 4.0.

Gene expression levels were quantified relative to the expression of β-actin for sample was calculated using the comparative 2^−ΔΔCT^ method. These data were subjected to analysis using the general linear models (GLM) procedure of SAS software (The SAS V9.0; SAS Institute, Cary, NC, USA). The statistical model included the main effect of age, line and all appropriate 2-way interactions. Differences among tissues or age were evaluated by Duncan’s test for multiple comparisons. The *p*-value for significance was set at *p* < 0.05.

Association analyses were carried out independently for SD02 and SD03 lines. The GLM procedure of SAS 9.0 was used to test association between the genotyped markers and laying traits. The following model was used:

(1) $${\text{Y}}_{\text{ij}}=\mu +{\text{L}}_{\text{i}}+{\text{G}}_{\text{j}}+{\text{L}}_{\text{i}}\times {\text{G}}_{\text{j}}+\text{e}$$

where Y is the traits measured on chickens, μ is the population mean, L_i_ is the fixed effect of lines, G_j_ is the fixed effect associated with genotype, (L_i_ × G_j_) is the interaction between line and genotype, and e is the random error. The values were presented as least square means ± standard error. The significance of least square means was tested with Duncan’s test (*p* < 0.01).

## 5. Conclusion

In summary, cloning the CRBP IV cDNA and characterization of the tissue distribution and ontogenic expression of the CRBP IV mRNA in Erlang mountainous chicken suggests that CRBP IV may play an important role in vitamin A metabolism. The mutation of c. 826T>C in the second exon was found to be associated with positive effects on egg production traits. The detected SNP may be useful for marker-assisted selection in chicken breeding programs.

## Figures and Tables

**Table 1:** Comparison of the amino acid sequence of Erlang mountainous chicken CRBP IV with other vertebrates.

Species	GenBank accession number	Amino acid Identity 1
*Gallus gallus*	XP_417606.2	98.4% (125/127)
*Sus scrofa*	NP_001138694.1	74.8% (95/127)
*Mus musculus*	NP_071303.1	72.4% (92/127)
*Rattus norveicus*	NP_001102163	71.7% (91/127)
*Homo sapiens*	NP_443192.1	70.9% (90/127)
*Pan troglodytes*	XP_001161299.1	70.9% (90/127)
*Bos taurus*	XP_871364.3	66.9% (85/127)
*Canis lupus familiaris*	XP_536740.1	57.5% (73/127)

1 % indicates amino acid sequence identity of others species to the Erlang mountainous chicken CRBP IV sequence.

**Table 2:** Genotypic and allelic frequencies at polymorphic site of CRBP IV gene among different lines.

Lines	*N.*	Alleles frequency		Genotypes frequency			*p* values of chi-square 1	*PIC*^2^
	
		T	C	TT	TC	CC		
SD02	188	0.641	0.359	0.399	0.484	0.117	0.471	0.354
SD03	161	0.630	0.370	0.373	0.515	0.112	0.182	0.357
Total	349	0.636	0.364	0.386	0.499	0.115	0.091	0.356

1 The test of Hardy-Weinberg Equilibrium. *p* < 0.05 indicates a significant deviation from Hardy-Weinberg Equilibrium.

2 PIC = polymorphism information content.

**Table 3:** Least square mean egg production traits, by genotype and lines of chicken CRBP IV gene.

Items		*N*	Traits					

			AFE	BWFE	FEW	TE300	HCLD	ALI
Genotypes	CC	40	169.3 ± 10.0 ^b^	1981.5 ± 296.5	40.4 ± 4.9	85.6 ± 20.4 ^a,A^	9.7 ± 3.8 ^a^	1.8 ± 1.2
	TC	174	175.1 ± 11.2 ^a^	1892.1 ± 244.5	39.9 ± 5.8	81.1 ± 16.9 ^b,A,B^	7.8 ± 3.6 ^b^	1.6 ± 0.7
	TT	135	176.5 ± 11.5 ^a^	1898.0 ± 247.9	40.5 ± 6.8	77.9 ± 18.2 ^c,B^	7.2 ± 3.2 ^b^	1.7 ± 0.8

Lines	SD02	188	169.8 ± 10.2 ^b^	1903.0 ± 241.2	40.2 ± 6.6	83.3 ± 18.4	7.7 ± 4.0	1.6 ± 0.9
	SD03	161	176.8 ± 10.9 ^a^	1919.5 ± 250.2	41.3 ± 5.3	81.3 ± 17.3	7.8 ± 4.5	1.5 ± 0.8

Values are shown as the least squares means ± standard error. Mean values marked by different letters within columns differ significantly (*p* < 0.05, lowercase letters ^a,b,c^) or highly significantly (*p* < 0.01, uppercase letters ^A,B^). AFE = age at first egg, BWFE = body weight at first egg, FEW = first egg weight, TE300 = total eggs for 300 days, HCLD = highest continuous laying days and ALI = average laying interval.

**Table 4:** Primer pairs were used in this study.

Primers	Primer Sequence (5′→3′)	Annealing Temperature (°C)	Product Length (bp)	Amplified Region
Primer pairs for measuring cloning and sequencing chicken CRBP IV gene				
*CRBPIV*-F	TGACTGTAACTCCTTCCCACAGTCT	56	464	3–466
*CRBPIV*-R	GAAGACAGCTTGCACCTTATGAC			

Primer pairs for measuring chicken CRBP IV gene expression				
*CRBPIV*-F	CATACCACAAGCACATTCAGAGA	58	125	1320–1444
*CRBPIV*-R	AGTTTGTCATTGTCCCAGGTAAC			
*β-actin*-F	GAGAAATTGTGCGTGACATCA	60	152	685–836
*β-actin*-R	CCTGAACCTCTCATTGCCA			

Primer pairs for screening chicken CRBP IV gene polymorphisms				
P1-F	CTTCCCACAGTCTAGCAA	56.6	236	3380706–3380919
P1-R	ATCAGCTAACAAATATCCTTC			
P2-F	TTCTTCTGTCAAAGGTATTG	55.2	244	3381431–3381654
P2-R	CTTCCTTCTGTAAGAACACAT			
P3-F	CAGAGCCTGGTTACCTG	56.6	180	3381893–3382052
P3-R	AGCTTGCACCTTATGACTACA			
